# Prevalence of burnout and predictive factors among oncology nursing professionals: a cross-sectional study

**DOI:** 10.1590/1516-3180.2020.0606.R1.1202021

**Published:** 2021-06-25

**Authors:** Bianca Sakamoto Ribeiro Paiva, Mirella Mingardi, Talita Caroline de Oliveira Valentino, Marco Antonio de Oliveira, Carlos Eduardo Paiva

**Affiliations:** I PhD. Researcher and Professor, Postgraduation, Hospital de Câncer de Barretos, Barretos (SP), Brazil.; II RN. Nurse and Master’s Student, Postgraduation, Hospital de Câncer de Barretos, Barretos (SP), Brazil.; III RN, MSc. Nurse and Doctoral Student, Postgraduation, Hospital de Câncer de Barretos, Barretos (SP), Brazil, Brazil.; IV BSc. Biostatistician, Learning and Research Institute, Hospital de Câncer de Barretos, Barretos (SP), Brazil.; V MD, PhD. Physician and Researcher, Postgraduation, Hospital de Câncer de Barretos, Barretos (SP), Brazil.

**Keywords:** Burnout, professional, Neoplasms, Occupational health, Oncology nursing, Personality, Occupational diseases, Burnout, Cancer, Quality of life for professionals

## Abstract

**BACKGROUND:**

Burnout is a syndrome that mostly affects professionals working in contact with patients and their caregivers. In oncology care, nursing professionals are constantly required to provide emotional support for patients and their caregivers, throughout the process of becoming ill, suffering and dying.

**OBJECTIVE:**

To evaluate the prevalence and factors associated with burnout in a sample of nursing professionals at a cancer hospital.

**DESIGN AND SETTING:**

Cross-sectional study conducted at Hospital de Câncer de Barretos.

**METHODS:**

The study population comprised 655 nursing professionals. Burnout syndrome was assessed using the Maslach Burnout Inventory Human Service Survey. Univariate analysis and binary logistic regression models were used to identify independent predictors associated with burnout.

**RESULTS:**

Among 304 nursing professionals included in the study, 27 (8.9%) were classified as presenting burnout according to the two-dimensional criteria, and four (1.3%) were classified based on the three-dimensional criteria. Workplace characteristics were not associated with burnout, while single marital status (odds ratio, OR = 2.695; P = 0.037), perceived workplace stressors, such as impatience with colleagues (OR = 3.996; P = 0.007) and melancholy (OR = 2.840; P = 0.021) were considered to be predictors of burnout. Nursing professionals who would choose the profession again (OR = 0.214; P = 0.001) were least likely to present burnout.

**CONCLUSION:**

Perceived workplace stressors are strongly associated with burnout. Strategies focusing on restructuring of daily work processes and on activities that stimulate positive relationships are important for professionals’ health because motivation to continue working in oncology nursing has a protective effect against burnout.

## INTRODUCTION

Oncological nursing is a specialty that can be characterized by constant exhaustion, especially emotional, due to the serious nature of cancer and the patient care profile. Nursing professionals within this specialty address patient suffering and death and perform the functions of encouraging and supporting family caregivers.^1^^,^^2^

In addition, these professionals’ practice can entail work overload, while maintaining institutional norms aimed at humanization and quality work. These factors require a high level of commitment from professionals and can lead to unsatisfactory interpersonal relationships in the work environment. All of these factors may cause professionals to develop burnout syndrome.^1^^,^^2^^,^^3^

Burnout syndrome is multifactorial and presents three distinct dimensions, defined as (1) emotional exhaustion (the basic dimension of individual stress, which causes professionals to feel overloaded and exhausted); (2) depersonalization (insensitivity or cynicism toward coworkers and patients); and (3) reduction of personal accomplishment (characterized by a sense of unproductiveness, lack of professional accomplishment and feelings of incompetence).^4^^,^^5^^,^^6^^,^^7^

Studies have shown that professionals and students in the field of healthcare have burnout levels that can be considered high in relation to those of other professions.^8^^,^^9^^,^^10^^,^^11^ A previous study by our research group found that 58.1% of physicians who work in oncology had two-dimensional burnout.^10^ In another study, we found that 44.9% of medical students also had levels compatible with two-dimensional burnout.^9^

Specifically, in nursing, a study conducted among nurses at six cancer centers showed that emotional exhaustion from burnout was present in more than 60%, while depersonalization was present in 28.2%. The study also found that the difficulty that these professionals had in helping patients cope with their illnesses was correlated with the burnout dimensions.^12^

Several studies have suggested that many oncology nurses present burnout or are at risk of this.^13^^,^^14^^,^^15^^,^^16^^,^^17^^,^^18^ These professionals are part of a specialty that has been recognized as the main clinical area that is exposed to emotional labor.^18^ Thus, it can be said that burnout is a matter of worldwide concern, which indicates that there is a need to improve the working conditions of professionals so that they can perform their functions with satisfaction, have good interpersonal relationships and consequently increase their productivity.

In addition, oncology nursing assists cancer patients and their families at all stages starting from diagnosis, including treatment, rehabilitation, dying, death and post-death. These professionals’ overburden of work is generated through the complexity and subtypes of the disease and the extension of care to the psychosocial environment. Therefore, understanding the factors associated with high levels of burnout among these professionals forms an essential component of healthcare practice in a philanthropic humanized hospital in a middle-income country.

## OBJECTIVE

The objective of this study was to evaluate the burnout levels of oncological nursing professionals and identify the factors that are related to burnout syndrome.

## METHODS

### Place of study

The Hospital de Câncer de Barretos (HCB), located in the city of Barretos, São Paulo, Brazil, is a public institution that is recognized as a national reference center for cancer treatment. Its hospital attends approximately 6,000 cancer patients daily, from all 27 Brazilian states, through the Brazilian National Health System, which guarantees full, universal and free access for the country’s population.^19^ It is a care, teaching and research institution and has three oncological units, for provision of various specialties for children, adults and elderly patients (Unit I), for children and adolescents (Children’s Unit) and for palliative care (Unit II). These three units have a total of 226 hospital beds, a multiprofessional team and both inpatient and outpatient services.

### Ethical aspects

This study was performed in accordance with the regulations of the Brazilian National Health Council (Conselho Nacional de Saúde Brasileiro), under its resolution no. 466/212, and was approved by the Research Ethics Committee of HCB (CEP/HCB no. 1.885.901; January 7, 2017). Nurses who voluntarily agreed to participate in the study provided their consent in writing.

### Study design

A descriptive cross-sectional study was conducted between June 2017 and September 2018.

### Study population and sample size

Nursing assistants, nurses and nursing coordinators working in Units I and II were included. Professionals who had been hired less than three months prior to the study were excluded.

In accordance with practices in Brazil and at the study site, nursing assistants are professionals with a technical level of education who are responsible for maintaining the patient’s hygiene, checking vital signs and administering medications. While nurses provide care directly to patients, plan the assistance and perform medium and high-complexity procedures, nursing coordinators do not have direct contact with patients. The latter are responsible for the bureaucratic and organizational functions of the staff and department.

The study population was composed of nursing assistants, nurses and nursing coordinators from among the total of 655 nursing professionals working in the oncology units (Unit I and Unit II).

### Procedures

Initially, informational posters about the research project were posted at strategic points in the oncology units to alert nursing professionals to the research event. Subsequently, the researchers invited all nursing assistants, nurses and nursing coordinators to participate in the study and attend meetings that were scheduled during work shifts in the outpatient, radiology, hospitalization, research and palliative care departments. At these meetings, the study was presented, questions were answered and all nursing professionals who were present were invited to participate in the study. At that time, those who agreed to participate in the study provided written consent and received the study questionnaires to answer. The evaluation questionnaires for this study were completed individually and confidentially by each person who had agreed to participate.

### Data collection

The following types of data were collected through the evaluation questionnaire:

- Sociodemographic data – age, gender, marital status, children, school education and other professional activity;
- Data on the professionals’ state of health – health problems and the professionals’ views of their own health, their own personality and whether they were a happy or unhappy person;
- Data on workplace characteristics – function, time of work, department, time dedicated to direct patient care and whether the work routine was exhausting;
- Data on activities outside of work – family meetings, leisure activities, physical activity, religion and influence of spirituality on work;
- Data on perceived workplace stressors – lack of recognition by the hospital, patients or relatives, difficulties in relationships among the nursing team or with multidisciplinary team members, excessive work, lack of time to perform other work activities, lack of resources for appropriate treatment of patients, institutional rules, lack of knowledge about the strategic planning of the hospital, lack of autonomy at work, constantly dealing with incurable and/or severe diseases, and feelings and symptoms in the work environment;
- Data on professional considerations – happiness with the professional activity, satisfaction with financial achievements, ability to perform professionally, feelings of importance to patients or coworkers, whether the individual would choose to be a nurse professional again, and satisfaction with professional evolution;
- The Maslach Burnout Inventory Human Services Survey (MBI-HSS) – This consists of 22 items that are answered using a seven-point Likert scale. Out of the total of 22 items, nine evaluate emotional exhaustion (EE domain; scored as low = 0-16; moderate = 17-26; high ≥ 27), five evaluate depersonalization (DP; low = 0-6; moderate = 7-12; high ≥ 13) and eight evaluate personal accomplishment (PA, low = 0-31; moderate = 32-38; high ≥ 39). The low, moderate and high scores for each dimension of burnout were obtained by summing the scores of the items in each dimension.^20^^,^^21^ The bidimensional criterion (high EE and DP scores) and the three-dimensional criterion (high EE and DP scores and low PA score) were used to identify burnout.^22^

The version of the MBI-HSS used in this study had previously been validated and adapted for use in the Portuguese language.^20^ The right to use this instrument was purchased from and authorized by Mind Garden, as described on the website http://www.mindgarden.com/.

The instruments used in the study were self-administered in paper format and were completed by the participants in an average of 20 minutes.

### Data analysis

The study population was characterized using frequency tables for qualitative variables and means and standard deviations for quantitative variables. Comparisons were made using the nonparametric Mann-Whitney test for continuous variables and the Pearson chi-square test or Fisher’s exact test for categorical variables. To identify independent predictors associated with burnout, variables with a P-value < 0.2 obtained in the univariate analysis were included in the binary logistic regression model. To compose the final model, we selected variables with a P-value < 0.05 (stepwise regression, Wald test). The IBM-SPSS software, version 21.0 (IBM Corp., Armonk, New York, United States) was used for statistical analysis, and the significance level was taken to be 0.05.

Missing values in the MBI-HSS were imputed by calculating the average of the responses for each item. Out of the total number of participants, 24 (7.9%) had at least one missing item in the MBI-HSS, and data allocation was used in these situations.

## RESULTS

### Sample description

Among the 655 nursing professionals potentially eligible to be invited to participate in the study , 11 (1.67%) did not agree to participate in the study, 139 (21.2%) were not approached for the study because they were absent from the department at the time of the meeting, 126 (19.2%) were unavailable on the date and at the time of the meeting and 74 (11.3%) were not included because they were participating in another study using the MBI-HSS instrument.

Thus, the response rate was 46.5%, i.e. 305 nursing professionals were included in this study. However, one participant did not respond to any item of the MBI-HSS and therefore was not included in the analyses relating to burnout.

### Demographic profile

The final sample of 305 nursing professionals consisted of 207 (67.9%) nurse assistants, 72 (23.6%) nurses and 26 (8.5%) nurse coordinators. The mean age was 36.0 years (standard deviation, SD = 9.1), and the mean duration of employment at the institution was 92.5 months. For 200 nursing professionals (67.1%), the daily work time dedicated to direct patient care was greater than 75%. In total, 279 (91.5%) of the participants were women, 184 (60.5%) were married or living as married and 192 (63.0%) had children; 193 (63.3%), 44 (14.4%) and 68 (22.3%) had technical, undergraduate education and postgraduate education levels, respectively (Table 1).

**Table 1.** Sociodemographic characteristics of nursing professionals working in oncology at a cancer hospital in Brazil (n = 305)

Variables	n (%)
**Age**	
Years, mean (SD)	36.0 (9.1)
**Gender**	
Female	279 (91.5)
Male	26 (8.5)
**Marital status**	
Married/living as married	184 (60.5)
Single	88 (28.9)
Separated/divorced/widowed	32 (10.5)
**Children**	
Yes	192 (63.0)
No	113 (37.0)
**Educational level**	
Technical	193 (63.3)
Graduate	44 (14.4)
Postgraduate	68 (22.3)
**Department**	
Outpatient	93 (30.8)
Hospitalization	85 (28.1)
Palliative care	56 (18.5)
Radiology	46 (15.2)
Research	22 (7.3)
**Function**	
Nursing assistants	207 (67.9)
Nurses	72 (23.6)
Nursing coordinators	26 (8.5)
**Length of time working at the institution**	
Months, mean (SD)	92.5 (68.2)
**Percentage of time dedicated to direct patient care**	
> 75% of working time	200 (67.1)
25%-75% of working time	61 (20.5)
< 25% of working time	37 (12.4)

Hours of daily work for nursing professionals (n = 305; 100%): 7 to 12 hours. SD = standard deviation.

### Burnout scores and prevalence

In total, 27 (8.9%) of the nursing professionals were identified as having two-dimensional burnout (high EE + high DP), and 4 (1.3%) were identified as having three-dimensional burnout (high EE + high DP + low PA).

**Figure 1.**
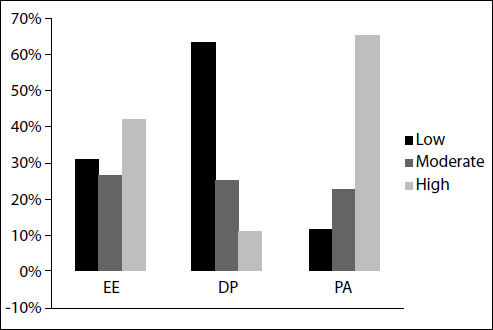
Burnout prevalence rate (%) among nursing professionals, in the different burnout domains and score categories. EE: emotional exhaustion; DP: depersonalization; PA: personal accomplishment. The scores are represented in columns of different shades: black, low levels; dark gray, moderate levels; light gray, high levels.

The scores for each dimension of burnout (EE, DP and PA) were categorized as low, moderate or high. Based on this categorization, high, moderate and low EE were present in 42.1%, 26.6% and 31.2% of the sample, respectively. High DP was present in 11.2%, and moderate and low DP in 25.3% and 63.5% respectively. For the dimension of PA, 11.8%, 22.7% and 65.5% of the sample presented low, moderate and high scores (Figure 1). The mean (with SD) burnout scores were 23.8 (12.1) for EE, 5.8 (5.5) for DP and 39.3 (7.2) for PA.

### Factors associated with bidimensional burnout

Table 2 shows that having children (65.0% versus 44.4%; P = 0.039) and considering oneself happy (77.8% versus 55.6%; P = 0.017), along with the majority of the professional consideration variables, were associated with low burnout.

**Table 2.** Variables associated with two-dimensional burnout in a sample of nursing professionals working in oncology at a cancer hospital in Brazil (n = 304)

Variable		Burnout		P-value
		No n (%)	Yes n (%)	
Participant characteristics				
Age in years median (minimum-maximum)		35 (20-72)	32 (21-50)	0.163^**^
Gender	Female	254 (91.7)	24 (88.9%)	0.715^*^
	Male	23 (8.3)	3 (11.1)	
Marital status	Married/living as married	173 (62.7)	11 (40.7)	0.090^*^
	Separated/divorced/widowed	29 (10.5)	3 (11.1)	
	Single	74 (26.8)	13 (48.1)	
Children	No	97 (35.0)	15 (55.6)	0.039
	Yes	180 (65.0)	12 (44.4)	
Educational level	Technical	175 (63.2)	17 (63.0)	0.839
	Graduate	41 (14.8)	3 (11.1)	
	Postgraduate	61 (22.0)	7 (25.9)	
Other professional activity	No	240 (87.3)	25 (92.6)	0.551 ^*^
	Yes	35 (12.7)	2 (7.4)	
Health problems	Not applicable	185 (67.5)	14 (51.9)	0.134
	Yes	89 (32.5)	13 (48.1)	
How do you consider your health?	Bad	64 (23.4)	10 (37.0)	0.158
	Good	209 (76.6)	17 (63.0)	
How do you consider your personality?	Pessimistic	77 (27.9)	10 (38.5)	0.263
	Optimistic	199 (72.1)	16 (61.5)	
Do you consider yourself a happy or unhappy person?	Unhappy	61 (22.2)	12 (44.4)	0.017
	Happy	214 (77.8)	15 (55.6)	
Workplace characteristics				
Function	Nursing assistants	187 (67.5)	19 (70.4)	1.000
	Nurses	66 (23.8)	6 (22.2)	
	Nursing coordinators	24 (8.7)	2 (7.4)	
Number of months working at the hospital (median (minimum-maximum))		72 (4-360)	72 (8-192)	0.471 ^**^
Department	Outpatient	84 (30.7)	9 (33.3)	0.105 ^*^
	Radiology	44 (16.1)	2 (7.4)	
	Hospitalization	72 (26.3)	13 (48.1)	
	Clinical research	22 (8.0)	0 (0.0)	
	Palliative care	52 (19.0)	3 (11.1)	
Percentage of time dedicated to direct patient care	< 25%	35 (12.9)	2 (7.7)	0.552
	25%-75%	57 (21.0)	4 (15.4)	
	> 75%	179 (66.1)	20 (76.9)	
Work routine	Not exhausting	25 (9.0)	1 (3.7)	0.490^*^
	Exhausting	252 (91.0)	26 (96.3)	
Perceived workplace stressors (no versus yes)				
Lack of recognition by the hospital		89 (32.1)	16 (59.3)	0.006
Lack of recognition by patients or relatives		25 (9.0)	8 (29.6)	0.004 ^*^
Difficulties in relationships among the nursing team		50 (18.1)	13 (48.1)	0.001
Difficulties in relationships with multidisciplinary team members		11 (4.0)	5 (18.5)	0.008 ^*^
Excessive work		122 (44.0)	19 (70.4)	0.014
Lack of time to perform other work activities		73 (26.4)	9 (33.3)	0.496
Lack of resources for appropriate treatment of patients		4 (1.4)	1 (3.7)	0.374 ^*^
Institutional rules		20 (7.2)	5 (18.5)	0.057 ^*^
Lack of knowledge about the strategic planning of the hospital		12 (4.3)	1 (3.7)	1.000 ^*^
Lack of autonomy at work		6 (2.2)	1 (3.7)	0.482 ^*^
Constantly dealing with incurable and/or severe diseases		51 (18.4)	7 (25.9)	0.440
Feelings at work	Sad/anxious	45 (16.2)	11 (40.7)	0.004^*^
	Discouraged	96 (34.7)	17 (63.0)	0.006
	No empathy with patients	3 (1.1)	3 (11.1)	0.010 ^*^
	Impatience with colleagues	26 (9.4)	9 (33.3)	0.001 ^*^
	Not respected	22 (7.9)	3 (11.1)	0.475 ^*^
	Unmotivated	79 (28.5)	18 (66.7)	< 0.001
	Not valued	76 (27.4)	13 (48.1)	0.029
	Not encouraged to improve as a professional	36 (13.0)	7 (25.9)	0.081 ^*^
	Dehumanized	17 (6.1)	5 (18.5)	0.034 ^*^
	Lack of interest from superiors or colleagues about the nurse’s opinions	22 (7.9)	7 (25.9)	0.008 ^*^
	Lack of interest from superiors or colleagues in relation to their professional capacity	25 (9.0)	4 (14.8)	0.307 ^*^
Symptoms in the work environment	Dizziness	121 (43.7)	14 (51.9)	0.425
	Tachycardia	118 (42.6)	13 (48.1)	0.578
	Tachypnea	13 (4.7)	2 (7.4)	0.631 ^*^
	Sweating	55 (19.9)	7 (25.9)	0.618
	Frequent headache	130 (46.9)	15 (55.6)	0.425
	Syncope	6 (2.2)	0 (0.0)	1.000 ^*^
	Overwhelming desire to cry	124 (44.8)	17 (63.0)	0.104
	Perception of professional impotence	81 (29.2)	14 (51.9)	0.019
	Melancholy	59 (21.3)	13 (48.1)	0.003
Professional considerations				
Happy with the professional activity	Not applicable	82 (29.8)	16 (59.3)	0.003
	Yes	193 (70.2)	11 (40.7)	
Satisfied with financial achievements	Not applicable	144 (52.2)	20 (74.1)	0.042
	Yes	132 (47.8)	7 (25.9)	
Able to perform professionally	Not applicable	66 (24.2)	13 (48.1)	0.011
	Yes	207 (75.8)	14 (51.9)	
Importance to patients	Not applicable	7 (2.5)	3 (12.0)	0.042 ^*^
	Yes	268 (97.5)	22 (88.0)	
Importance to coworkers	Not applicable	38 (13.8)	8 (29.6)	0.044 ^*^
	Yes	238 (86.2)	19 (70.4)	
Would choose to be a nurse professional again	Not applicable	51 (18.5)	14 (53.8)	< 0.001
	Yes	225 (81.5)	12 (46.2)	
Satisfied with professional evolution	Not applicable	93 (33.7)	13 (50.0)	0.131
	Yes	183 (66.3)	13 (50.0)	
Activities outside of work				
Family meetings	Not applicable	161 (58.1)	22 (81.5)	0.022
	Yes	116 (41.9)	5 (18.5)	
Leisure activities	Not applicable	234 (84.5)	25 (92.6)	0.395 ^*^
	Yes	43 (15.5)	1 (7.4)	
Physical activity	Not applicable	182 (65.9)	20 (74.1)	0.522
	Yes	94 (34.1)	7 (25.9)	
Religion	Not applicable	14 (5.1)	1 (3.7)	0.999 ^*^
	Yes	263 (94.9)	26 (96.3)	
Influence of spirituality in work	Not applicable	90 (32.8)	9 (33.3)	0.999
	Yes	184 (67.2)	18 (66.7)	

Pearson’s chi-square test; ^*^Fisher’s exact test; ^**^Mann-Whitney test.

Regarding workplace characteristics, no statistically significant association with burnout was observed. In evaluating the presence of perceived workplace stressors, there was higher prevalence of burnout related to lack of recognition by the hospital (P = 0.006), difficulties in relationships among the nursing team (P = 0.001) and lack of recognition by patients or their relatives (P = 0.004). In addition, the professionals who reported feeling the following, due to the work process, were more susceptible to burnout: sad or anxious (40.7%; P = 0.004); discouraged (63%; P = 0.006); unempathetic with patients (11.1%; P = 0.010); impatient with colleagues (33.3%; P = 0.001); unmotivated (66.7%; P < 0.001); not valued (48.1%; P = 0.029); treated without humanization (18.5%; P = 0.034); that their superiors or colleagues were uninterested in their opinions (25.9%; P = 0.008); professionally impotent (51.9%; P = 0.019); or melancholy (48.1%; P = 0.003) (Table 2).

### Multivariate analyses

The adjusted multivariate model showed that the nursing professionals who would choose to enter the nursing profession again (odds ratio, OR = 0.214; P = 0.001) had a lower probability of being diagnosed with burnout. In contrast, feeling impatient with colleagues (OR = 3.996; P = 0.007) or melancholy (OR = 2.840; P = 0.021) and being single (OR = 2.695; P = 0.037) were independently associated with a greater likelihood of burnout (Table 3).

**Table 3.** Binary logistic regression analysis on the potential factors associated with two-dimensional burnout (Barretos Cancer Hospital, n = 304)

Burnout				P-value
Variable	Category		OR (95% CI)	
Marital status	Married/living as married		1	------
	Separated/divorced/widowed		2.483 (0.586-10.511)	0.217
	Single		2.695 (1.061-6.844)	0.037
Environmental stressor factors	Impatient with colleagues	No	1	------
		Yes	3.996 (1.470-10.864)	0.007
	Melancholy	No	1	------
		Yes	2.840 (1.168-6.905)	0.021
Daily work variables	I would choose to be a nurse professional again	No	1	------
		Yes	0.214 (0.087-0.526)	0.001

Binary logistic regression analysis. P-value < 0.05. OR = odds ratio; CI = confidence interval.

## DISCUSSION

This study evaluated the prevalence of burnout among active oncology nurses at a Brazilian hospital and the potential factors related to the syndrome. Approximately 9% and 1.3% of the participants presented two-dimensional and three-dimensional burnout, respectively. Impatience with colleagues, melancholy and being single were the factors related to greater risk of burnout syndrome. Furthermore, we found that the participants who reported that they would choose to enter the nursing profession again presented lower risk of burnout.

The results shown in the present study have been identified in the worldwide literature.^6^^,^^12^^,^^13^^,^^14^^,^^15^^,^^16^^,^^17^^,^^18^ The high level of burnout among oncology nurses and a growing lack of job satisfaction might negatively affect their quality of life and have an impact on the quality of nursing care and the services to be provided in general.^17^^,^^23^ A study conducted in hematology and oncology clinics and palliative care units in three different public hospitals in Turkey demonstrated high emotional exhaustion scores among nurses who perceived that their interpersonal relationships were bad and who were not satisfied with workplace. Their emotional exhaustion was higher than that of nurses who were satisfied. In addition, a positive correlation between job satisfaction scores and personal accomplishment scores was identified.^17^

The results from that study in Turkey by Yıldırım and Kocatepe^17^ supported the notion that burnout decreases as job satisfaction increases. Furthermore, this can be interpreted as denoting that emotional exhaustion is the most important component of burnout status. In this context, the present study showed that approximately 42% and 27% of the sample had high and moderate EE, respectively. It is noteworthy that Brazilian oncological nurses are more exhausted, considering that EE is the main cause and the initial symptom of burnout syndrome. However, this finding contradicts the data from a quantitative, observational, cross-sectional multi-center study that was conducted in Spain among oncology nurses. A total of 101 oncology nurses were included and 19.2% and 38.4% were found to have high and moderate EE, respectively. Another important finding was that high DP was more prevalent in Spain (21.1%) than in Brazil (11.2%). Low PA was evident in 65.5% of the sample of the present study and in 45.5% of the Spanish professionals, thus showing that there were lower levels of personal fulfillment among the Brazilian nurses.^24^

A meta-analysis study included a total sample of 9,959 nurses from oncology services. The prevalence of EE was 30% and that of low PA was 35%. Thus, the presence and risk of burnout among these employees worldwide are considerable. This needs to be identified and institutional measures need to be implemented to prevent associated conditions.^25^

Factors that have been correlated with two-dimensional burnout were mostly present in the workplace. For example, there was a lack of recognition of nurses’ work among patients or family members (P = 0.004). Healthcare professionals dedicate considerable time to integral care of patients and their relatives, especially in place of study.

The main philosophy of nursing is humanized care, according to one of the guidelines of the Brazilian National Health System. However, humanized care and attention has been a one-way street from professionals to patients, and professionals do not always receive the attention and care that they should. Another factor that may contribute to burnout is the professionals’ view of their work effort as being for the benefit of patients, often without taking self-care into account, or attention to their limits in relation to work, which leads to psychological distress.^26^ This type of stressor, known as overwork, was evident (P = 0.014) in the present study.

The institution’s humanization philosophy allows healthcare professionals to have a closer relationship with patients and family members, which can be a protective factor for both patients and workers. On the other hand, this philosophy requires the development of emotional skills to address the emotional excess or burden that results from providing daily care for patients and family caregivers. Thus, nursing professionals are almost always exposed to the stressors of the work environment, which may impair their work-related quality of life.

Oncology is a specialty that requires much from professionals, especially emotionally. This has been identified as the largest clinical field in which nurses are exposed to emotional labor. Nurses in this field are in greater contact with suffering and death than are colleagues in other areas.^18^ Constantly coping with serious life-threatening illness generates feelings associated with burnout, such as discouragement (P = 0.006), lack of empathy with patients (P = 0.010), lack of patience with coworkers (P = 0.001), lack of motivation (P < 0.001) and impotence (P = 0.019). Oncology nurses are faced with diseases that generate suffering and that often have an outcome of death. This causes frailty and a feeling of impotence in professionals, because there is no possibility of reversing the situation.^1^ Education and training for dealing with death, and discussion of attitudes towards death, can be a way to decrease the levels of burnout among oncology nurses.^15^

In the binary logistic regression analysis, it could be seen that separated, divorced and widowed individuals (OR = 2.483) and single individuals (OR = 2.695) were more likely to develop burnout than were married individuals. This finding corroborates other studies conducted in Australia and China.^14^^,^^27^ The emotional support and stability that a family or partner can offer are important protective factors that support mental health and prevent burnout. In addition, it is understood that social support in its different forms is considered predictive of and protective against burnout syndrome.^28^

Another important result from the present study was that relationship difficulties among the nursing team were associated with burnout. In Brazil, nursing is further subdivided into categories. It is a hierarchy, with different positions, functions and salaries. This scenario may not be healthy for relationships among professionals and may cause difficulty and imbalance in the relationships between team members.

The professionals who reported feeling a lack of patience with coworkers (OR = 3.996; P = 0.007) were approximately four times more likely to experience burnout than those who did not report this feeling. One dimension of burnout, i.e. depersonalization, corroborates this finding. The main characteristics of this domain are cynicism and insensitivity toward coworkers, patients and family members, thus indicating that burnout itself leads to a lack of patience with colleagues, which further increases the probability of developing burnout.^5^ The feeling of melancholy at work (OR = 2.840) increases the risk of burnout, compared with professionals who do not feel melancholy. It is evident that depression is related to burnout: melancholy is a common feeling among depressive individuals, since it is characterized by mental fatigue.^29^

The professionals were asked whether they would choose to enter the nursing profession again (OR = 0.214), and those who said yes had a lower probability of burnout than those who said no. This finding demonstrates that achievement and job satisfaction are protective factors against burnout.

This study had some limitations. The first was that it was a cross-sectional study, and it was therefore impossible to determine cause-and-effect relationships. The second was that we evaluated work stressors based on the opinions of nursing professionals and did not objectively measure their numbers of appointments or actual working time. However, we believe that perceptions of one’s work, and not necessarily the work itself, are more important with regard to the genesis of burnout. Thirdly, the data were based on nursing professionals who were working in a single oncology center, and this may limit the generalizability of our results to other care settings or may not reflect the overall reality of Brazil. Fourthly, the sample consisted mostly of women. Thus, this reflected the demographics of this field, which can be explained by the historical context within which the profession emerged. Although an increasing number of men are entering the profession, women still comprise the majority of nurses in this country. Additionally, the questionnaire that was developed to obtain sociodemographic data relating to nursing professionals’ health, perceived stressors in daily work and activities outside of work had not been validated.

## CONCLUSIONS

An important number of nursing professionals working in oncology were identified as having possible burnout. The association between perceived workplace stressors and burnout suggested that organizational dynamics had contributed to creation of a stressful work environment that affected these professionals’ emotional wellbeing and commitment to the field. In this context, strategies for reorganizing work processes and practices that promote professional interaction, involvement in decision-making and sharing of emotions are relevant for self-management, health promotion and maintenance of care quality.

## References

[1] Cumbe VFJ. (2010). Síndrome de Burnout em Médicos e Enfermeiros Cuidadores de Pacientes com Doenças Neoplásicas em Serviços de Oncologia [Dissertation].

[2] Davis S, Lind BK, Sorensen C. (2013). A comparison of burnout among oncology nurses working in adult and pediatric inpatient and outpatient settings. Oncol Nurs Forum.

[3] Hecktman HM. (2012). Stress in pediatric oncology nurses. J Pediatr Oncol Nurs.

[4] Maslach C, Jackson SE. (1981). The measurement of experienced burnout. Journal of Organizational Behavior.

[5] Maslach C, Schaufeli WB, Leiter MP. (2001). Job burnout. Annu Rev Psychol.

[6] Meneghini F, Paz AA, Lautert L. (2011). Occupational factors related to burnout syndrome components among nursing personnel. Texto & Contexto Enfermagem.

[7] Yates M, Samuel V. (2019). Burnout in oncologists and associated factors: A systematic literature review and meta-analysis. Eur J Cancer Care (Engl).

[8] Dyrbye L, Shanafelt T. (2016). A narrative review on burnout experienced by medical students and residents. Med Educ.

[9] Boni RADS, Paiva CE, de Oliveira MA (2018). Burnout among medical students during the first years of undergraduate school: Prevalence and associated factors. PLoS One.

[10] Paiva CE, Martins BP, Paiva BSR. (2018). Doctor, are you healthy? A cross-sectional investigation of oncologist burnout, depression, and anxiety and an investigation of their associated factors. BMC Cancer.

[11] Mladen S, Loughan A, Kinser P, Crawford M, Jones A, Edwards S, Rybarczyk B, Braun SE. (2019). An Analysis of Psychological Distress Profiles and their Correlates in Interdisciplinary Health-care Professional Students. Glob Adv Health Med.

[12] Emold C, Schneider N, Meller I, Yagil Y. (2011). Communication skills, working environment and burnout among oncology nurses. Eur J Oncol Nurs.

[13] Wu S, Singh-Carlson S, Odell A, Reynolds G, Su Y. (2016). Compassion Fatigue, Burnout, and Compassion Satisfaction Among Oncology Nurses in the United States and Canada. Oncol Nurs Forum.

[14] Guo YF, Luo YH, Lam L, Cross W, Plummer V, Zhang JP. (2018). Burnout and its association with resilience in nurses: A cross-sectional study. J Clin Nurs.

[15] Guo Q, Zheng R. (2019). Assessing oncology nurses’ attitudes towards death and the prevalence of burnout: A cross-sectional study. Eur J Oncol Nurs.

[16] Gribben L, Semple CJ. (2020). Factors contributing to burnout and work-life balance in adult oncology nursing: An integrative review. Eur J Oncol Nurs.

[17] Yıldırım D, Kocatepe V. (2020). A Comparison of Burnout and Job Satisfaction among Cancer Nurses in Oncology, Hematology and Palliative Care Clinics. Psychiatr Danub.

[18] Zaghini F, Biagioli V, Proietti M (2020). The role of occupational stress in the association between emotional labor and burnout in nurses: A cross-sectional study. Appl Nurs Res.

[19] Sistema Único de Saúde (SUS) (1990). B. Lei n^o^ 8.080, de 19 de setembro de 1990. Brasília.

[20] Tamayo MR. (1997). Relação entre a Síndrome de Burnout e os valores organizacionais no pessoal de enfermagem de dois hospitais públicos [Dissertation].

[21] Moreira D de S, Magnago RF, Sakae TM, Magajewski FR. (2009). Prevalência da síndrome de burnout em trabalhadores de enfermagem de um hospital de grande porte da Região Sul do Brasil [Prevalence of burnout syndrome in nursing staff in a large hospital in south of Brazil]. Cad Saude Publica.

[22] Campos JA, Maroco J. (2012). Adaptação transcultural Portugal-Brasil do Inventário de Burnout de Maslach para estudantes [Maslach Burnout Inventory - Student Survey: Portugal-Brazil cross-cultural adaptation]. Rev Saude Publica.

[23] Russell K. (2016). Perceptions of Burnout, Its Prevention, and Its Effect on Patient Care as Described by Oncology Nurses in the Hospital Setting. Oncol Nurs Forum.

[24] De la Fuente-Solana EI, Gómez-Urquiza JL, Cañadas GR (2017). Burnout and its relationship with personality factors in oncology nurses. Eur J Oncol Nurs.

[25] Cañadas-De la Fuente GA, Gómez-Urquiza JL, Ortega-Campos EM (2018). Prevalence of burnout syndrome in oncology nursing: A meta-analytic study. Psychooncology.

[26] de Paiva LC, Canário ACG, de Paiva China ELC, Gonçalves AK. (2017). Burnout syndrome in health-care professionals in a university hospital. Clinics (Sao Paulo).

[27] Poulsen MG, Poulsen AA, Khan A, Poulsen EE, Khan SR. (2015). Recovery experience and burnout in cancer workers in Queensland. Eur J Oncol Nurs.

[28] Velando-Soriano A, Ortega-Campos E, Gómez-Urquiza JL (2020). Impact of social support in preventing burnout syndrome in nurses: A systematic review. Jpn J Nurs Sci.

[29] Mendes ED, Viana TC, Bara O. (2014). Melancholy and depression: a psychoanalytic study. Psicologia: Teoria e Pesquisa.

